# LLaMAC: low-cost biosignal sensor based large multimodal dataset for affective computing

**DOI:** 10.1038/s41597-025-06165-4

**Published:** 2025-12-04

**Authors:** Chang-Gyu Lee, Joo Young Kim

**Affiliations:** Culture Wellness R&D Group, Korea Institute of Science and Technology, Gyeonggi-do, Republic of Korea

**Keywords:** Aggression, Bioinformatics

## Abstract

The LLaMAC dataset was developed to predict the success of audio-visual media via emotion prediction. It was created using low-cost biosignal sensors, with emotional questionnaires in both continuous (valence, arousal, dominance) and discrete domains (emotion type and intensity: neutral, fun, sadness, anger, fear), and included over 100 participants. Questionnaires on liking and familiarity were also collected. The dataset contains five biosignals—EEG, GSR, PPG, SKT, and RESP—and seven questionnaire measures. Biosignals were validated using statistical metrics and signal-to-noise ratios, while questionnaire responses were assessed with scatter plots and statistical analyses. Emotion classification was performed using a Light Gradient Boosting Machine (LightGBM). The dataset enables biosignal-based prediction of emotions and liking, correlation analysis between continuous and discrete emotions, and investigation of biosignal differences related to familiarity, which can further inform emotion and liking predictions.

## Background & Summary

For those who produce audio-visual media, such as movies, dramas, over-the-top media services (OTT), musical shows, and performances, the emotional state of the viewers is an important factor. Because the emotional state of the viewers affects the success of the audio-visual media^1^. This means that emotion prediction provides significant benefits to producers of the audio-visual media^2^. Therefore, various studies were conducted to predict the emotional states of the viewers, through facial expressions^3,4^, gestures^3,4^, and voices^3^. Recently, researches on predicting viewers’ emotional states from viewers’ biosignals were actively conducted^5^. In order to conduct this research, however, a dataset consisting of biosignals and emotional states is necessary. As shown in Table 1, therefore, many datasets were introduced consisting of **biosignals** and **emotional states** of people who **watching audio-visual media**. Among them, DREAMER^2^, ASCERTAIN^6^, AMIGOS^7^, Emognition^8^, and EmoWear^9^ created dataset using low-cost biosignal sensors, and AMIGOS^7^, Emognition^8^, MAHNOB-HCI^10^, FACED^11^, and Yang *et al*.,^12^ created dataset by using both continuous and discrete domains for emotional survey. FACED^11^ and G-REx^13^ created dataset by performing experiments with more than 100 subjects, and ASCERTAIN^6^, AMIGOS^7^, EmoWear^9^, FACED^11^, and DEAP^14^ created dataset by including a liking and a familiarity in survey. However, there is no dataset that uses low-cost biosignal sensor, performs survey in both continuous and discrete domains, conducts experiments with more than 100 subjects, and performs survey in both liking and familiarity. Therefore, this paper introduces a dataset that satisfying these conditions. The importance of each condition will be explained below.

**Table 1:** Comparison between LLaMAC and previous datasets.

Name of dataset	Low cost sensors	Survey both continuous and discrete emotions	Large scale participants	Survey both liking and familiarity
LLaMAC	O	O	O	O
DREAMER^2^	O	—	—	—
ASCERTAIN^6^	O	—	—	O
AMIGOS^7^	O	O	—	O
Emognition^8^	O	O	—	—
EmoWear^9^	O	—	—	O
MAHNOB-HCI^10^	—	O	—	—
FACED^11^	—	O	O	O
Yang *et al*.,^12^	—	O	—	—
G-REx^13^	—	—	O	—
DEAP^14^	—	—	—	O
DECAF^17^	—	—	—	—
WESAD^30^	—	—	—	—
CASE^33^	—	—	—	—
SEED-V^31^	—	—	—	—

First, it is important to create a dataset using low-cost biosignal sensors, because translational value requires that emotion prediction works with the types of devices consumers will actually use. The number of channels, measurement range, and resolution generally varies depending on the price of the biosignal sensor. When technology to predict emotions using biosignals becomes commercialized, users will use low-cost biosignal sensors, or in other words, consumer-grade biosignal sensors. The criteria for defining low-cost sensors are as follows. First, their price is significantly lower than that of medical-grade devices, thereby improving accessibility for researchers and developers. Second, they have been explicitly described as low-cost or consumer-grade biosignal sensors in prior literature. Of course, the low-cost biosignal sensors mentioned here mean low-cost biosignal sensors that have performance suitable for emotion prediction, such as Emotiv Epoc-X^2^. For this reason, previous researches have used low-cost biosignal sensors, such as Emotiv Epoc-X, NeuroSky MindWave, Muse 2, Empatica E4, Shimmer ECG, and Shimmer GSR^2,6–9^. Therefore, considering the commercialization of the technology, it is reasonable to create a dataset using low-cost biosignal sensors, which ensures both experimental validity and real-world scalability.

Second, it is important to create a dataset by surveying both continuous and discrete emotions, because translationally relevant models must operate across different theoretical and practical representations of affect. Emotional response data can be structured into continuous and discrete domains in affective computing. The continuous model captures affect along dimensional axes such as valence, arousal, and dominance^15^, while the discrete model categorizes emotions into basic types such as joy, anger, or sadness. The selection of these categories was grounded in the basic emotions framework^16^, which identifies a set of universally recognizable emotions that exhibit distinct expressive, physiological, and behavioral signatures. Continuous domain emotion has the advantage of being able to describe whole emotion without being affected by differences in language expression of emotion^2,10^. On the other hand, discrete domain emotion is a long standing approach that has the advantage of being easier to understand intuitively about emotion^2^. The reason for conducting the emotion survey for both continuous and discrete domains was to enable biosignal-based emotion prediction for continuous or discrete domains, and additionally to identify the relationship between continuous and discrete domain emotions. Previous researches have also conducted surveys in both domains^7,8,10–12^. Therefore, it is reasonable to conduct emotion surveys in both domains, thereby enhancing the dataset’s flexibility for real-world applications that may demand either categorical or dimensional outputs.

Third, it is important to create a dataset that conducts an experiment with more than 100 participants, since translational relevance also depends on robustness across diverse users. Such datasets gain statistical power as the number of participants increases. For this reason, previous researches have conducted experiments on large scale participants^11,13^. Therefore, it is reasonable to conduct an experiment with a large number of people to create a dataset enabling the development of predictive models that can generalize across heterogeneous populations rather than being limited to narrow laboratory cohorts.

Finally, it is important to create a dataset that surveys liking and familiarity, since translational value requires accounting for ecological factors that shape real-world emotional responses. Liking asks whether participants like the audio-visual media they viewed, and familiarity asks whether participants have viewed the audio-visual media before the experiment. As mentioned earlier, this dataset was created to check the state of people watching an audio-visual media. For audio-visual media producers, not only the emotions of the audience but also their liking are important factors. This is because liking is a more direct evaluation of the audio-visual media than emotion. Additionally, whether the audio-visual media has already been seen is also an important factor, because participants may not feel the same tension when watching a horror movie they have already seen. If there is a difference in biosignals, classification should be performed considering this difference. For this reason, previous researches have included liking and familiarity in their questionnaires^6,7,9,11,14^. Therefore, it is reasonable to conduct a survey that includes liking and familiarity, which anchors the dataset in ecologically valid user experiences.

## Methods

This section includes a description of the ethical procedures for protecting participants, the experimental environment, pre-experimental prohibitions, demographics of participants, audio-visual media for emotion elicitation, used biosignal sensors to be worn, emotion questionnaire, and the experimental protocol and procedures.

### Ethics statement

The research background and purpose, research period, participant requirements, recruitment method, consent acquisition method, research method, biosignal sensors, analysis method, safety evaluation criteria and evaluation method, expected adverse effects/cautions and solutions for these, experimental pause and dropout criteria, participants’ risks and benefits, and safety and privacy protection methods of this experiment were reviewed by the institutional review board (IRB) of the Korea institute of industrial technology (KITECH) with an application number of 2024-004-002. The board did not provide a consent waiver.

### Experimental environment

**Fig. 1:**
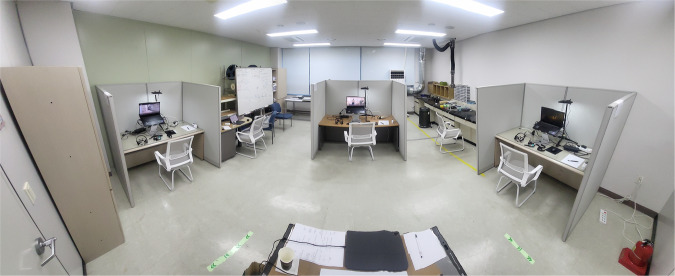
Experimental space.

As shown in Fig. 1, there were three separate desks and chairs in the experimental space to block the surrounding view and to make an isolated environment. This is to prevent visual distraction from other participants and surrounding environments^2^. Audio-visual media was played, biosignals were acquired, and surveys were conducted via a laptop installed on the desk. The visual media was presented on a 17.3-inch laptop monitor and the audio media was presented through a wired earphones. This was to prevent auditory disturbances caused by other participants and surrounding environments. By constructing identical and independent desks and using individual earphones, three participants in the same experimental space were able to conduct the experiment without disturbing each other. Finally, an air conditioner was installed in the experimental space to prevent changes in temperature and humidity in the experimental space, and the desired temperature was maintained at a constant of 22 °C. This is taken into consideration because body temperature and skin conductivity can change depending on air temperature and humidity.

All biosignals in the dataset were recorded through a single master computer, with all devices directly synchronized to its system clock, which also served as the source for all timestamps. This design inherently prevents inter-controller misalignment and temporal drift, ensuring clock-level synchronization across all signals without the need for additional validation.

### Affective stimuli

The previous researches used audio-visual media for emotion elicitation, and some of them used self-generated audio-visual media^14,17–22^. However, most of these audio-visual media were in English. It was difficult to use these audio-visual media for the general Korean public who are not fluent in English. Therefore, the authors of this paper created audio-visual media using Korean trailers and some scenes from movies, dramas, and documentaries. We prepared a set of 20 audio-visual media candidates for each target emotion. A pilot study involving 97 participants, who did not participate in the main experiment, was conducted to independently assess the emotional elicitation effectiveness of these audio-visual media. Participants reported their emotional responses using both continuous ratings and discrete labels. Based on these results, we excluded media that exhibited high rate of cross-emotional misattribution, where participants consistently responded with a different emotion than intended. For example, media designed to induce anger but frequently rated as sadness were removed. The number of remaining candidates varied by emotion depending on the degree of misattribution observed. From these validated candidates, we selected a final set of 10 audio-visual media for each emotion, aiming to preserve within-category diversity while minimizing potential bias from manual selection. This process ensured a balance between emotional clarity and representative variation, making the dataset suitable for training robust and generalizable affective models.

As a result, a total of 50 audio-visual media were prepared, with 10 audio-visual media for each of the five emotions: neutral, fun, sadness, anger, and fear. Information about each audio-visual medium is shown in Table 2. And the length of each audio-visual media was the same, 60 seconds. This is because audio-visual media of 1 to 10 minutes in length can trigger emotions^20,23,24^, and audio-visual media of such a short length prevents various emotions from being triggered or becoming habitual^10^.

**Table 2:** Information about audio-visual media.

Intended emotion	Title	Description
Neutral	None	Full moon screen and insect sounds
	None	Wood burning screen and sound
	None	Wood carving screen and sound
	None	Wood burning screen and sound
	None	Sunset seascape and sound of waves
	None	A screen showing a shooting star falling from the night sky
	None	Aurora screen and dreamy sound in the night sky
	None	Screen and sound of swaying reeds
	None	Daytime seascape and waves sound
	None	Bamboo Forest Screen and Sound
Fun	Extreme job	Narcotics investigation team runs chicken restaurant for undercover investigation
	Midnight runners	A story about two police academy students who get caught up in a kidnapping case
	OK! Madam	Couple involved in plane hijacking on first trip abroad
	Honest candidate	The campaign of a congressional candidate who can no longer lie
	Saturday night live Korea	Characteristics of middle-aged women who use smartphones
	Jexi	Artificial intelligence intervenes excessively in actors’ daily lives
	Confidential assignment	Investigation of cooperation between South and North Korea
	Architecture 101	Advice on first love and first kiss
	The spy: Undercover operation	A spy encounters his wife during an operation
	Daddy you, daughter me	The story of a father and daughter switching bodies
Sadness	National geographic	Mother elephant leaves her dead baby elephant behind
	The most beautiful goodbye	A family’s story of saying goodbye to a mother with terminal cervical cancer
	The hospiece	Daily life in a hospice hospital where cancer patients are admitted
	Be with you	Husband and child reunited with dead wife
	Mom’s Post-it	Post-it notes left by my dead mother while she was alive
	The notebook	A story about a couple who broke up and reunited and stayed together until old age
	Dear my friends	Conversation between an elderly woman with dementia and her friends
	You are my sunshine	A conversation between a man and his lover who are in prison with AIDS
	My lovely angel	The story of a man who accidentally became the guardian of a deaf and blind child
	Still alice	A story about a mother with Alzheimer’s and her family
Anger	Silenced	Sexual assault incident at a school for the hearing impaired
	My golden kids	The appearance of a middle school student who is violent at home
	The discloser	Exposing and covering up corruption in the military
	Norigae	Sexual harassment scandal revealed through actress’ death
	Spirits’ homecoming	Stories of ‘Comfort Women’ during Japanese occupation of Korea
	Another promise	Legal battle with big business over industrial accident
	New trial	Retrial of a person who was falsely accused and served time in prison
	Broken	Father seeks revenge on the murderer of his daughter
	News	Pedestrian kicks pregnant cat, causing miscarriage
	The penthouse: War in life	A perpetrator who speaks confidently about school violence
Fear	Annabelle: Creation	The story of a doll with a soul
	GONJIAM: Haunted asylum	A horror experience at the closed Gonjiam Psychiatric Hospital
	Insidious: The red door	The family’s horrible nightmare involving the red doo
	Hereditary	A curse left by a deceased mother to her family
	The autopsy of Jane Doe	Jane Doe’s autopsy reveals horrifying secret
	The 8th night	A fight to prevent the release of the ghost’s seal
	Ghost station	Strange things happening in the subway station
	The nun	The evil spirit that dwells in the convent
	Hush	A story of a deaf woman fighting off attackers in a remote cottage
	The visit	Strange and creepy things happen to siblings when they visit their grandparents’ house

### Biosignal sensors

Three biosignal sensors were used to acquire five biosignals from the participants. The specific specifications of each sensor are shown in Table 3. The Emotiv Epoc-X is a low-cost electroencephalogram (EEG) sensor suitable for affective computing^2^, and it was used to generate the DREAMER^2^ and AMIGOS^7^ datasets. EEG sensor such as the Biosemi Active II (used in the MAHNOB-HCI and DEAP datasets^10,14^) cost over 12,000 EUR, and the DSI-24 (Yang *et al*.^12^) costs at least 22,000 USD, whereas the Emotiv Epoc-X, employed in the DREAMER dataset and recognized as a low-cost device, is priced at approximately 1,000 USD. The Empatica E4 is a low-cost galvanic skin response (GSR), photoplethysmograph (PPG), and skin temperature (SKT) sensor, which is a practical and valid tool for studying heart rate or heart rate variability in stationary conditions^25^. It is an effective tool as much as the Biopac MP150 in emotion recognition tasks^26^ and has been validated in emotion recognition tasks^8,27–29^. While the Biosemi Active II system was used for blood flow, skin conductance, and body temperature measurements in MAHNOB-HCI and DEAP^10,14^ (≥12,000 EUR), the Empatica E4, applied in Emognition and WESAD^8,30^ and introduced as a consumer-grade biosignal sensor, costs about 1,700 USD. For respiration (RESP), the Biosemi Active II (≥12,000 EUR) was replaced with the GDX-RB, which is available for approximately 125 USD.

**Table 3:** Specification of biosignal sensors.

Sensor	Biosignal	Specification
Emotiv, Epoc-X	EEG	Channel: 14
		Sampling rate: 256 Hz
		Resolution: 0.1275 *μ*V
		Bandwidth: 0.16–43 Hz
		Dynamic range: 8400 *μ*V
		Data transfer: Bluetooth
Empatica, E4	GSR,	Sampling rate (GSR): 4 Hz
		Resolution (GSR): 900 pS
		Range (GSR): 0.01–100 *μ*S
	PPG,	Sampling rate (PPG): 64 Hz
		Resolution (PPG): 0.9 nW/Digit
	SKT	Sampling rate (SKT): 4 Hz
		Resolution (SKT): 0.02 °C
		Range (SKT): −40–115 °C
		Accuracy (SKT): 0.2 °C within 36–39 °C
		Data transfer: Bluetooth
Vernier, GDX-RB	RESP	Sampling rate: 10 Hz
		Resolution: 0.01 N
		Range: 0–50 N
		Data transfer: USB-A

### Self-assessment questionnaire

After watching the prepared audio-visual media, the participants filled out a seven-item questionnaire. It included the continuous domain emotion (valence, arousal, dominance), the discrete domain emotion (type and intensity of emotion: neutral, fun, sadness, anger, and fear), liking for the audio-visual media, and whether the audio-visual media had been seen (familiarity).

First, the continuous domain emotion consists of three items: valence, arousal, and dominance, and the participants answered with a number between 1 and 5. This questionnaire has been used in many previous studies investigating emotions^2,7,9,10,12,14,17^ since it was proposed by Russell^15^. Among them, valence refers to the intrinsic positivity or negativity of an emotion. A high-valence emotion is pleasant, while a low-valence emotion is unpleasant. Next, arousal indicates the intensity or activation level of an emotion, ranging from calm (low) to highly excited (high). Finally, dominance describes the degree of control or power an individual feels over a situation or emotion, ranging from submissive (low) to dominant (high).

Second, the discrete domain emotion consists of two items: emotion type and intensity, and participants were asked to express one emotion among neutral, fun, sadness, anger, and fear, and to rate the intensity of that emotion with a number between 1 and 6.

Third, participants expressed their liking for audio-visual media by choosing between disliking, neutral, and liking. As mentioned in Affective stimuli section, the affective stimuli consisted of trailers and scenes from movies, dramas, and documentaries. Participants were asked to select ‘liking’ if they wanted to watch the movie or the entire audio-visual media after watching the prepared 60-second audio-visual media, or ‘disliking’ if they did not want to watch the movie or the entire audio-visual media. This can be used to perform biosignal-based liking prediction for audio-visual media.

Fourth, participants answered whether they had seen or not seen the audio-visual media used in the experiment. Participants were asked to select ‘seen’ if they had seen the edited 60-second audio-visual media, the original audio-visual media, or the movie/drama/documentary of the corresponding audio-visual media, or ‘not seen’ if they had not seen any of it.

### Experimental procedure and protocol

Before the experiment, the participants were given two prohibitions. One was not to drink alcohol the day before the experiment and the other was not to consume caffeine on the day of the experiment. These two things were prohibited because they affect the cardiovascular system. Such prohibitions are common in researches measuring biosignals^8,30^.

**Fig. 2:**
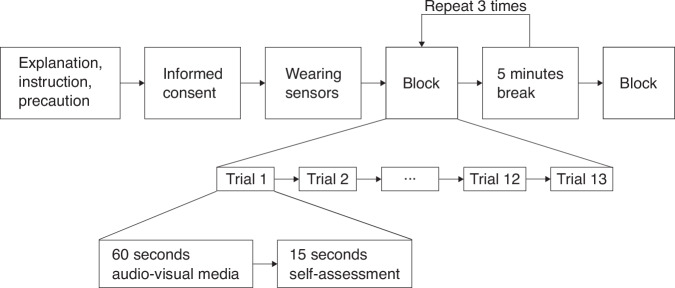
Experimental procedure.

Participants appeared in the experimental space at the appointed time. Depending on the recruitment of participants, as few as one and as many as three participants conducted the experiment together. As shown in Fig. 2, the overall experimental process is structured in three hierarchical levels. The first row represents the pre-experimental explanation, consent form signing, sensor placement, four experimental blocks, and three inter-block breaks. The second row details the structure within each block, consisting of 12 or 13 trials. The third row illustrates the trial sequence, where each trial involved 60 seconds of audio-visual media viewing followed by at least 15 seconds of self-assessment.

After all recruited participants appeared, the contents of the experiment were introduced. This included the research background and purpose, participant requirements, conditions for ineligibility for participation, biosignal sensors to be worn, the experimental task, the experimental procedure, the experimental time, expected adverse effects/cautions and solutions for these, reward, reasons for reward restrictions, voluntary participation and withdrawal from the experiment, and safety and privacy protection methods. The experimental task was to survey emotions after viewing 50 audio-visual media, and it was emphasized that the survey was about elicited emotions, not intended emotions from audio-visual media. The experiment took about 150 minutes, and the reward for participating in the experiment was about $35.

**Fig. 3:**
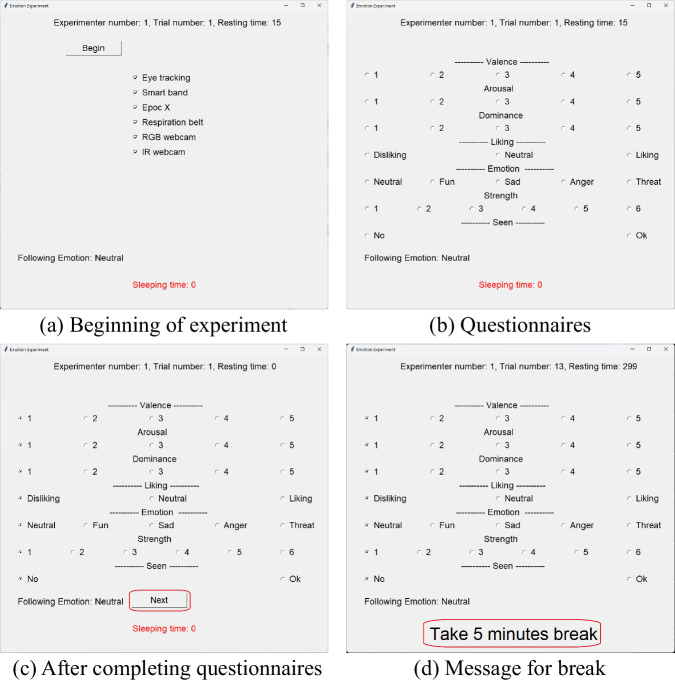
GUIs for experiments.

Then, the graphical user interface (GUI) of the experiment shown in Fig. 3 was introduced. First, as shown in the upper part of Fig. 3a, the trial number was presented. This was to provide information about the progress of the experiment. The participant started the experiment by pressing the ‘Begin’ button of Fig. 3a. Then, a 60-second audio-visual media was played. The participant was asked to look only at the monitor while the audio-visual media was played. This is because there may be participants who do not faithfully perform the task of feeling emotions while looking at the audio-visual media.

After the audio-visual media was played, the participants filled out the questionnaire of Fig. 3b. As in the DREAMER dataset experiment^2^, the meaning of each item was explained to participants so that they could understand each questionnaire, then all items were explained with three specific examples. Participants were asked to spend at least 15 seconds (resting time) filling out the questionnaire as shown in the upper right part of Fig. 3b. This was to force them to restore from the elicited emotion to a neutral state. Such survey time limitation was common in previous studies^6,17,31^. While a short 15-second interval was applied between successive trials within the same emotional category, the experiment was structured to minimize strong emotional contrast by presenting stimuli of the same category consecutively (e.g., strong fun followed by mild fun). In addition, when transitioning between different emotional categories—particularly between distant ones such as fun and sadness—we implemented an extended recovery phase. This consisted of a 5-minute break and the presentation of at least two neutral stimuli, ensuring a minimum interval of 7 minutes and 30 seconds before the next emotion-inducing stimulus. After completing the questionnaire, as shown in the lower part of Fig. 3c, a ‘Next’ button appeared on the screen, which allowed them to start the next trial. In addition, as shown in lower part of Fig. 3c, the intended emotion of the next trial was presented by ‘following emotion’. In the absence of this information, participants could have experienced fear while viewing neutral audio-visual media, after viewing consequtive fear-inducing audio-visual media. This was also to prevent participants from being startled when watching audio-visual media that induces fear.

While viewing 50 audio-visual media, three 5-minute breaks were given. Breaks were intended to keep participants focused on the experiment and prevent physical and mental fatigue. Breaks began after viewing the 13th, 26th, and 38th audio-visual media. Instructions for this break were provided as shown in the lower part of Fig. 3d. During the break, the Emotiv Epoc-X was removed. This was because wearing the Emotiv Epoc-X for a long time could cause pain in the scalp. After the break, the ‘Next’ button appeared in the same way, allowing the next trial to begin.

Then, the participant heard a description of the sleeping time shown in the lower part of Fig. 3a,b,c. This was a value that increased by one if the participant closed their eyes for more than 5 seconds, and the closing of the eyes was identified through Tobii Pro Spark. Participants were dropped out when their sleeping time reached 3. This was done to prevent participants from losing focus on the experiment. The experiment ended after viewing all 50 audio-visual media.

Finally, the participant was told again about the precautions. First, they should not doze off or fall asleep. Second, they should not move their head, non-dominant hand, and body. Last, they should not touch any of the biosignal sensors they were wearing.

**Fig. 4:**
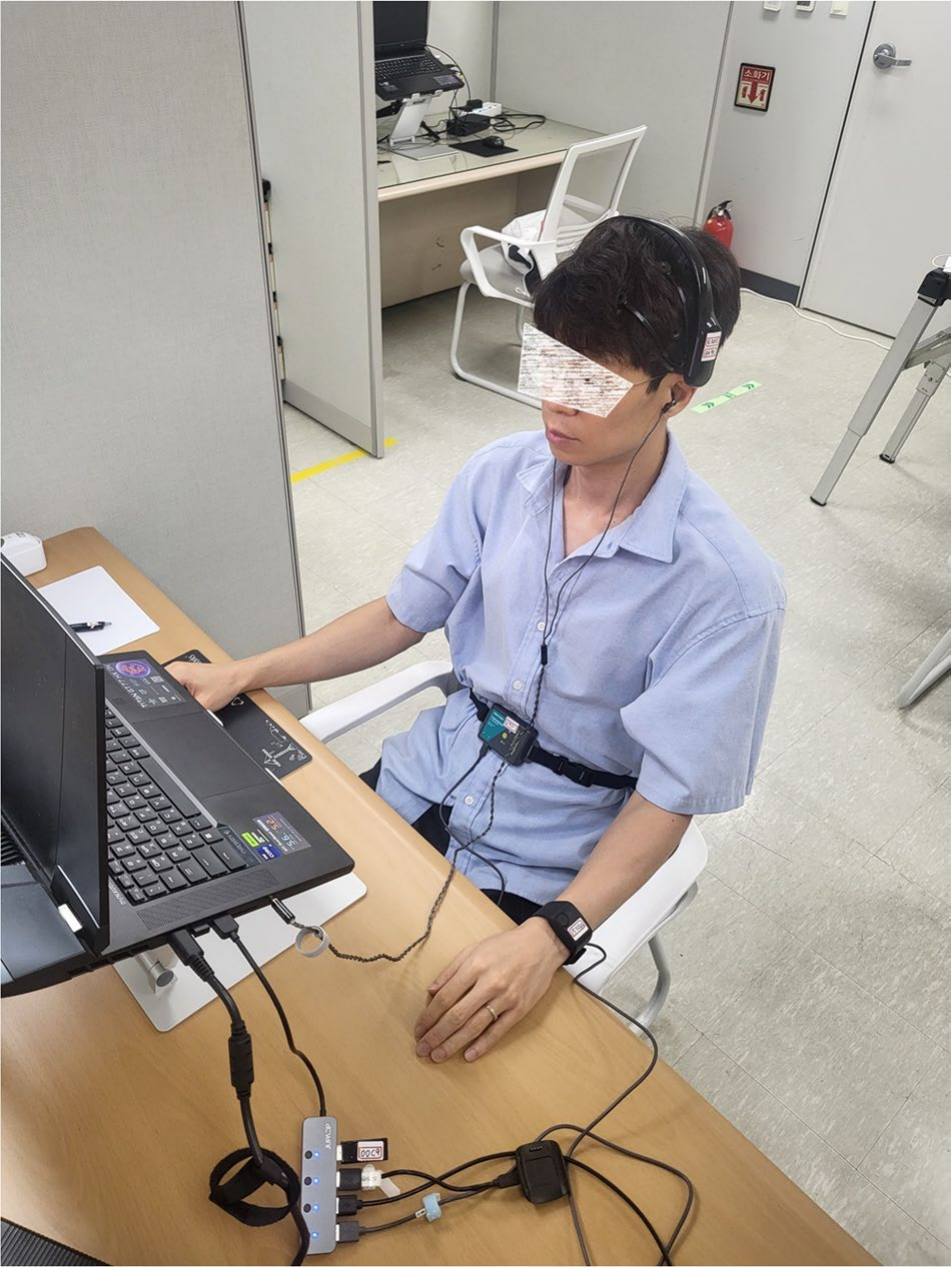
Participant wearing biosignal sensors.

After hearing this explanation, participants signed a consent form indicating that they wanted to participate in the experiment. This consent also includes consent to share their data in an anonymized form. Then, the participants were asked to turn off all smart devices, including smartphones, smartwatches, and smartbands. This is because notifications generated from smart devices interfere with the participant’s task. Then, his or her body temperature was measured and the biosignal sensors were worn. As shown in Fig. 4, the Empatica E4 was worn on the non-dominant hand, the Emotiv Epoc-X was worn on the head according to the 10-20 system, and the Vernier GDX-RB was worn on the abdomen or chest. Then, the participant was instructed not to move his or her non-dominant hand and head, and then the second temperature measurement was performed. At this time, the two temperature measurements were performed to confirm that the participant had reached a normal body temperature. There was a time gap of approximately 30 minutes between the two temperature measurements. If the participant’s body temperature changed by more than 1 °C, the body temperature was measured again after 5 minutes. Once the body temperature was determined to be normal, the experiment was started.

**Fig. 5:**
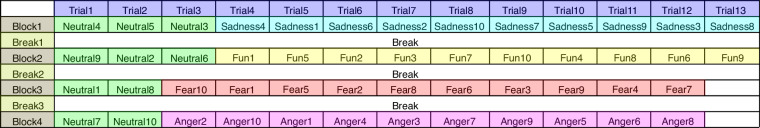
One example for the presentation order of 50 prepared audio-visual media.

It is also important in what order the prepared audio-visual media are presented to the participants. This will be explained with an example in Fig. 5. The experiment consisted of four blocks, each of which contained 13, 13, 12, and 12 trials. At this time, audio-visual media eliciting neutral emotions were presented in the first 3, 3, 2, and 2 trials of each block. This was to remove daily dependencies^2^, to acquire normal-state biosignals, and to recover from the elicited emotions^31^. In the next 10 trials for each block, audio-visual media eliciting the same emotion were played sequentially, with the emotions were selected randomly. The reason for grouping audio-visual media of the same emotion into one block is that it is difficult for participants to experience sudden emotional changes. This block level experimental protocol was similar to that of previous dataset experiments^11,12^. In addition, the 10 prepared audio-visual media for each emotion were also presented in a fully randomized order. At this time, the fully randomized presentation order for same emotion included audio-visual media that elicited neutral emotion. In summary, the presentation order of emotions was fully randomized at the block level, and the presentation order of audio-visual media was fully randomized at the trial level.

### Participants

A total of 114 people participated in the experiment, and their mean age was 27.73, standard deviation (SD) was 9.08, and they ranged from 20 to 57 years old. Of them, 57 were male and 57 were female. However, the data of six of them were invalid. The reasons were as follows: 1. One participant consumed caffeine on the day of the experiment. 2. One participant moved continuously during the experiment. 3. One participant withdrew from the experiment due to fear of audio-visual media that elicited fear. 4. One participant’s data was missing due to a sensor malfunction. 5. One participant did not understand Korean fluently. All audio-visual media were prepared in Korean, incorporating dialogue, narration, and culturally specific references. The excluded participant reported difficulty in comprehending the content during the post-experiment debriefing, indicating that limited Korean language proficiency hindered their engagement with the stimuli. Because proper comprehension of the media was essential for eliciting reliable emotional responses and maintaining internal validity, we excluded this participant’s data. 6. One participant was found to have a tic disorder, so the data was excluded.

## Data Records

The dataset is available at ‘LLaMAC: Low-cost Biosignal Sensor based Large Multimodal Dataset for Affective Computing’^32^. In this dataset, each folder represents a participant number, and each folder contains the files answer.csv, band_#.csv, eeg_#.csv, and respiration_#.csv, where # represents the trial number that each participant performed in the experiment. Trial numbers range from 1 to 50.

1. answer.csv

The first column of this file represents the trial number, and the second column represents the number of audio-visual media presented to the participant. The numbers of each audio-visual media are in the same order as they appear in the Table 2. Columns 3 to 5 show the results of the continuous domain emotion questionnaire on a 5-point scale, measuring valence (1 = negative to 5 = positive), arousal (1 = calm to 5 = highly excited), and dominance (1 = submissive to 5 = dominant). Column 6 shows the results of the liking questionnaire (1: disliking, 2: neutral, 3: liking). Column 7 (1: neutral, 2: fun, 3: sadness, 4: anger, 5: fear,) and column 8 reflects the strength of that emotion on a 6-point scale (1 = weakest, 6 = strongest).column 8 (ranges from 1 to 6) show the results of the discrete domain emotion questionnaire, and the last column 9 shows the results of the seen (1: unseen, 2: seen) questionnaire.

2. band_#.csv

This file contains raw GSR, PPG, and SKT data. Columns 1 and 2 show the raw GSR values (*μ*S) and the time at which they were acquired, columns 3 and 4 show the raw PPG values and the time at which they were acquired, and finally columns 5 and 6 show the raw SKT values (°C) and the time at which they were acquired. At this time, all times used UNIX time. Some participants’ GSR signals showed values outside the measurable range of the sensor. This is due to the non-contact nature of the electrodes. Therefore, it is recommended not to use the GSR signals of participants 32, 37, 40, 47, 54, 55, 56, 70, and 99. Additionally, we recommend excluding specific trials of participants listed in Table 4 for the same reasons.

**Table 4:** Biosignals, participant numbers, and trial numbers recommended for exclusion.

Biosignal	Participant number	Trial number
GSR	7	27, 28, 36
	19	18
	59	35
	111	1
EEG	20	26
	59	13
	89	13
	105	38, 50
	107	38

3. eeg_#.csv

The first column of this file is the EEG signal acquisition time, which is presented in UNIX time. Next, from the second to the fifteenth columns are raw 14-channel EEG signals (*μ*V) of AF3, F7, F3, FC5, T7, P7, O1, O2, P8, T8, FC6, F4, F8, and AF4. Due to missing some EEG signals, we recommend excluding certain trials of certain participants as shown in Table 4.

4. respiration_#.csv

The first column of this file presents the time at which the respiration sensor acquired the signal in the format year-month-day hour:minute:second.microsecond. The second column is the raw signal (N) measured by the belt-type respiration sensor.

### Biosignal processing

Heart rate (HR) was estimated from the PPG signal by first centering the raw signal via median subtraction to remove baseline fluctuations. A Butterworth bandpass filter (0.5 - 8 Hz) was applied to isolate cardiac-related frequencies and reduce noise and motion artifacts. Peaks corresponding to heartbeats were detected using an adaptive peak detection algorithm with a minimum peak distance of 0.35 s and a prominence fraction of 0.08, after which inter-beat intervals (IBIs) were calculated and converted to beats per minute (BPM). Heart rate variability (HRV) features, including the mean and standard deviation of IBIs, the root mean square of successive differences (RMSSD), and pNN50 (IBI threshold of 0.05 s), were also derived. In addition, the temporal slope and zero-crossing rate (ZCR) were computed to characterize trend and fluctuation dynamics.

RESP signals were processed similarly: an adaptive peak detection algorithm with a minimum peak distance of 1.0 s and a prominence fraction of 0.08 was used to identify breaths, followed by calculation of inter-breath intervals (IBIs) and conversion to breaths per minute (BPM). with additional measures including the mean and standard deviation of IBIs, the temporal slope, and the ZCR.

GSR signals were interpolated to address missing values and analyzed in terms of both tonic and phasic components. Phasic activity was characterized by the number of skin conductance responses (SCRs) per trial, along with their mean and maximum amplitudes. Tonic activity was summarized using robust descriptive statistics (mean, variance, interquartile range, and quantiles) as well as the linear slope. In addition, the ZCR was calculated to capture fluctuation dynamics.

SKT signals were represented through descriptive statistics, temporal slope, and interquartile range, providing insights into both baseline levels and gradual thermal drifts over the course of the trials.

EEG signals were recorded using the Emotiv Epoc-X headset, which employs 14 saline-based electrodes (AF3, F7, F3, FC5, T7, P7, O1, O2, P8, T8, FC6, F4, F8, AF4) referenced to CMS/DRL electrodes that implement active common-mode noise rejection. Device-level preprocessing included a 0.2-45 Hz band-pass filter, 50/60 Hz notch filters, and a 5th-order sinc digital filter. As these filters are applied by the device, no additional software-based band-pass or notch filtering was performed. Independent component analysis (ICA) was not used for the removal of ocular (typically  < 4 Hz) or electromyographic (typically  > 20 Hz) artifacts. Instead, corrupted or missing trials and channels were excluded following a trial-level quality control procedure (see Table 4). This strategy ensured that subsequent analyses were conducted on minimally processed but quality-controlled EEG signals.

Feature extraction was then applied to the retained EEG data. In the time domain, features included robust descriptive statistics (mean, variance, skewness, kurtosis, interquartile range, quantiles, root mean square (RMS), and energy), Hjorth parameters (activity, mobility, complexity), line length, zero-crossing rate, and temporal slope. In the frequency domain, Welch’s method is used to estimate the power spectral density (PSD), from which absolute and relative band powers were calculated for *δ* (1-4 Hz), *θ* (4-7 Hz), *α* (8-13 Hz), *β* (13-30 Hz), and *γ* (30-45 Hz). Additional spectral features included spectral entropy and alpha peak frequency. Trials with insufficient valid samples produce NaN values and were excluded from aggregate analyses.

## Technical Validation

This section validated the generated data. This included validation of audio-visual media, biosignals and self-assessment.

### Validation for audio-visual media

To evaluate the effectiveness of this selection procedure, we compared emotion induction performance between the initial, non-selected pool and the refined set. As shown in Table 5, the refined set achieved higher precision for most emotions, with particularly large improvements for anger (from 0.705 to 0.893) and fear (from 0.887 to 0.936), which are often confounded in affective computing due to their shared high-arousal, low-valence characteristics. Cohen’s *κ* improved from 0.682 to 0.753, indicating stronger agreement between intended and perceived emotions.

**Table 5:** Precision and agreement (Cohen’s *κ*) of emotion induction across stimulus.

Emotion	Non-selected	Selected
Neutral	0.620	0.618
Fun	0.856	0.896
Sadness	0.756	0.809
Anger	0.705	0.893
Fear	0.887	0.936
Cohen’s *κ*	0.682	0.753

**Fig. 6:**
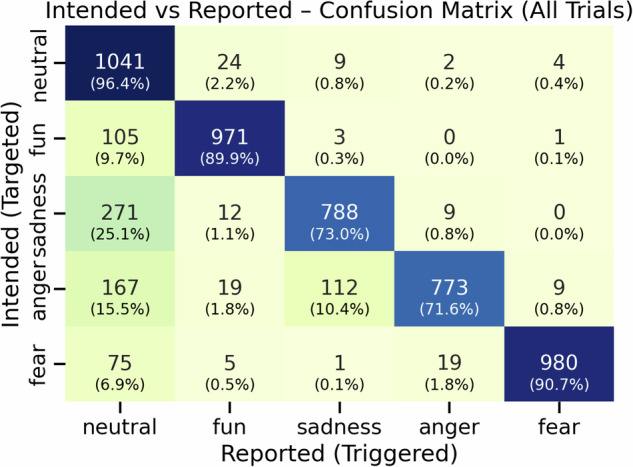
Self-reported elicitation outcomes for emotion-eliciting videos: intended vs. reported confusion matrix.

While pilot data validated the initial selection of stimuli, their effectiveness was further assessed in the main experiment with 108 participants, yielding 5,400 self-assessment responses. Precision for each discrete emotion was computed, and overall performance metrics showed strong agreement between intended and reported emotions, with overall accuracy of 0.843 and Cohen’s *κ* of 0.804 (see Table 6). To evaluate stimulus-level performance, intended labels were compared with participant-reported labels, and a confusion matrix displaying both raw counts and row-normalized percentages was generated (see Fig. 6). The diagonal percentages in the matrix show the elicitation rate of each video, clearly indicating how effectively each stimulus elicited its target emotional response.

**Table 6:** Main experiment elicitation validity: precision and accuracy between intended and reported emotions.

Emotion	Precision	Accuracy
Neutral	0.627	0.878
Fun	0.942	0.969
Sadness	0.863	0.923
Anger	0.963	0.938
Fear	0.986	0.979

### Validation for biosignals

As shown in Tables 7, 8, 9 and 10, to validate the quality of recorded biosignals, statics and signal-to-noise ratios (SNRs) were computed. SNRs were computed using an autocorrelation-based center fitting technique, with quadratic polynomial interpolation performed over four samples (excluding the center, including two samples on either side). This SNR computation has also been used in previous studies^8,9^. SNR values were computed for each biosignal, each trial, and each participant. The mean, SD, maximum, Q75, Q50, Q25, Q0.3, and minimum of the SNR values obtained for each biosignal are shown in Tables 9 and 10. Here, Q75, Q50, Q25, and Q0.3 represent the top 75%, 50%, 25%, and 0.3% SNR values, respectively. As shown in Tables 9 and 10, the mean of the SNR values ranges from 11.42 dB to 43.37 dB, and the SD ranges from 0.50 dB to 2.37 dB. Additionally, the proportion of SNR values less than 5 dB and 10 dB for each biosignal is shown in Tables 9 and 10.

**Table 7:** Statistics of the EEG.

	AF3	F7	F3	FC5	T7	P7	O1	O2	P8	T8	FC6	F4	F8	AF4
Mean	4329.426	4324.187	4318.127	4337.318	4316.220	4320.330	4317.076	4310.385	4298.251	4283.354	4455.416	4473.891	4373.373	4416.178
SD	96.150	91.210	69.468	67.147	74.103	71.645	69.558	77.715	103.816	101.351	87.844	82.029	94.299	103.355
Maximum	7858.718	7910.769	7882.949	7779.359	7732.692	7723.077	7994.487	8184.231	8112.180	8351.025	7911.410	8173.974	8384.615	8120.641
Minimum	531.026	589.744	588.974	590.385	606.026	518.462	581.026	60.128	600.000	298.333	432.821	507.308	616.282	384.487

**Table 8:** Statistics of GSR, SKT, HR, and RESP.

	GSR	SKT	HR	RESP
Mean	1.577	33.334	69.706	17.679
SD	3.972	1.966	6.005	3.624
Maximum	44.143	37.950	91.667	38.940
Minimum	0.010	24.970	48.658	6.216

**Table 9:** Statics of the SNRs for EEG.

	AF3	F7	F3	FC5	T7	P7	O1	O2	P8	T8	FC6	F4	F8	AF4
Mean	43.47	43.48	43.49	43.48	43.48	43.49	43.49	43.49	43.47	43.46	43.47	43.48	43.48	43.47
SD	0.80	0.80	0.78	0.79	0.82	0.78	0.77	0.78	0.82	0.85	0.80	0.79	0.81	0.81
Maximum	47.04	46.05	46.08	46.06	46.07	45.98	45.99	46.03	45.85	45.89	45.96	46.00	45.95	46.35
Q75	43.70	43.69	43.67	43.67	43.66	43.66	43.66	43.67	43.67	43.67	43.68	43.67	43.70	43.69
Q50	43.63	43.63	43.63	43.63	43.63	43.63	43.63	43.63	43.63	43.63	43.63	43.63	43.64	43.63
Q25	43.56	43.59	43.60	43.60	43.60	43.61	43.61	43.60	43.60	43.59	43.58	43.59	43.57	43.55
Q0.3	40.14	40.35	40.33	40.17	39.76	40.48	40.51	40.51	40.34	39.72	40.10	40.35	40.28	40.03
Minimum	39.57	37.58	39.57	39.62	37.63	39.50	39.51	39.46	36.05	35.73	39.18	39.15	37.60	39.34
Proportion of SNR values below 5 dB (%)	0.00	0.00	0.00	0.00	0.00	0.00	0.00	0.00	0.00	0.00	0.00	0.00	0.00	0.00
Proportion of SNR values below 10 dB (%)	0.00	0.00	0.00	0.00	0.00	0.00	0.00	0.00	0.00	0.00	0.00	0.00	0.00	0.00

**Table 10:** Statics of the SNRs for GSR, SKT, HR, and RESP.

	GSR	SKT	PPG	RESP
Mean	37.14	37.37	17.88	26.84
SD	0.84	0.27	2.32	2.46
Maximum	40.37	38.40	31.10	33.33
Q75	37.46	37.55	19.30	28.68
Q50	37.24	37.36	17.74	27.43
Q25	36.98	37.19	16.32	25.52
Q0.3	31.55	36.65	12.56	18.60
Minimum	16.59	36.45	9.93	15.33
Proportion of SNR values below 5 dB (%)	0.00	0.00	0.00	0.00
Proportion of SNR values below 10 dB (%)	0.00	0.00	0.02	0.00

All biosignals except GSR, HR, and RESP showed values greater than 21.47 dB, and 99.98 %, 99.98 %, and 75.04 % of GSR, HR, and RESP values showed values greater than 10 dB, respectively.

### Validation for self-assessments

**Fig. 7:**
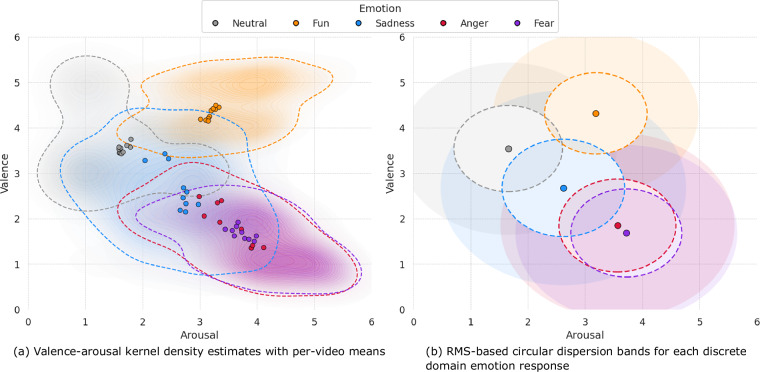
Valence-arousal distribution from self-assessment.

Figure 7 shows the valence and arousal distribution from self-assessment. Figure 7a shows 2D kernel density estimates (KDE) for each emotion with overlaid mean points per video, highlighting skewness, tails, and multimodal patterns to emphasize non-Gaussian features. Figure 7b summarizes each emotion using pooled means and RMS-based circular dispersion bands, which replace error bars and explicitly account for low or variable valence-arousal correlations. Additionally, 95% covariance ellipses were computed to support the main findings. Together, the KDE, circular bands, and covariance ellipses provide a clearer and more accurate visualization of the valence-arousal distributions across all emotions.

Next, statistical analysis was conducted to validate that participants elicited different emotions depending on the intended emotion. One-way analysis of variance (ANOVA) was used for the analysis, with the intended emotion of audio-visual media as the independent variable and valence, arousal, and dominance as the dependent variables. As a result, there were statistically significant differences in valence [F(4, 5395) = 3017.682, p  < 0.001***], arousal [F(4, 5395) = 1044.064, p  < 0.001***], and dominance [F(4, 5395) = 447.357, p  < 0.001***] depending on the intended emotion. Additionally, a post-hoc pairwise comparison with Tukey’s honestly significant difference (HSD) test showed that there was no statistically significant difference between the dominances of fun and anger (p = 0.372). On the other hand, there was a statistically significant difference between all other comparisons (p  < 0.001***).

Additionally, a Chi-square test was conducted to assess the congruence between the intended emotions of the audio-visual media and the reported discrete emotions from the 5,400 self-assessments. The analysis revealed a statistically significant association between the two variables [*χ*^2^(16) = 14824.090, p  < 0.001***]. These results indicate a strong association between participants’ reported discrete emotions and the intended emotion of the audio-visual media, supporting the validity of the emotional classifications in the dataset.

Also, the distribution of continuous domain emotion responses based on the participants’ discrete domain emotion responses is shown in Fig. 7b. This distribution is intended to confirm the correlation between continuous and discrete domain emotions. The valence and arousal distribution by audio-visual media in Fig. 7a was similar to the valence and arousal distribution by discrete domain emotion responses in Fig. 7b. The valence and arousal distribution for the discrete domain emotion responses of neutral, fun, sadness, anger, and fear were [high valence / low arousal], [high valence / high arousal], [low valence / low arousal], [low valence / high arousal], and [low valence / high arousal], respectively.

Next, statistical analysis was performed to confirm whether the differences in valence, arousal, and dominance according to each discrete domain emotion response showed statistically significant differences. Discrete domain emotion responses were used as independent variables and valence, arousal, and dominance were used as dependent variables. As a result, statistically significant differences were found in all of valence [F(4, 5395) = 4197.515, p  < 0.001***], arousal [F(4, 5395) = 1328.217, p  < 0.001***], and dominance [F(4, 5395) = 720.434, p  < 0.001***]. Additionally, a post-hoc pairwise comparison with Tukey’s HSD test showed that there was no statistically significant difference between the valences of anger and fear (p = 0.981) and between the arousals of anger and fear (p = 0.542). On the other hand, there was a statistically significant difference between the dominances of anger and fear (p = 0.002**) and all other comparisons (p  < 0.001***).

### Emotion classification

A baseline emotional classification task was implemented to evaluate whether the biosignals could predict emotional states. A total of 574 features were extracted from EEG, GSR, PPG, SKT, and RESP. These comprised time-domain statistical features (minimum, maximum, mean, variance, standard deviation, median, interquartile range, Q10 and Q90 values, skewness, kurtosis, and coefficient of variation) and amplitude/energy-related features (root mean square and energy). In addition, biosignal-specific features were extracted: EEG frequency-domain characteristics (band power in Delta, Theta, Alpha, Beta, Gamma), GSR (slope and zero-crossing rate), PPG (heart rate and mean inter-beat interval), SKT (slope-based features), and RESP (breaths per minute and mean inter-breath interval).

**Fig. 8:**
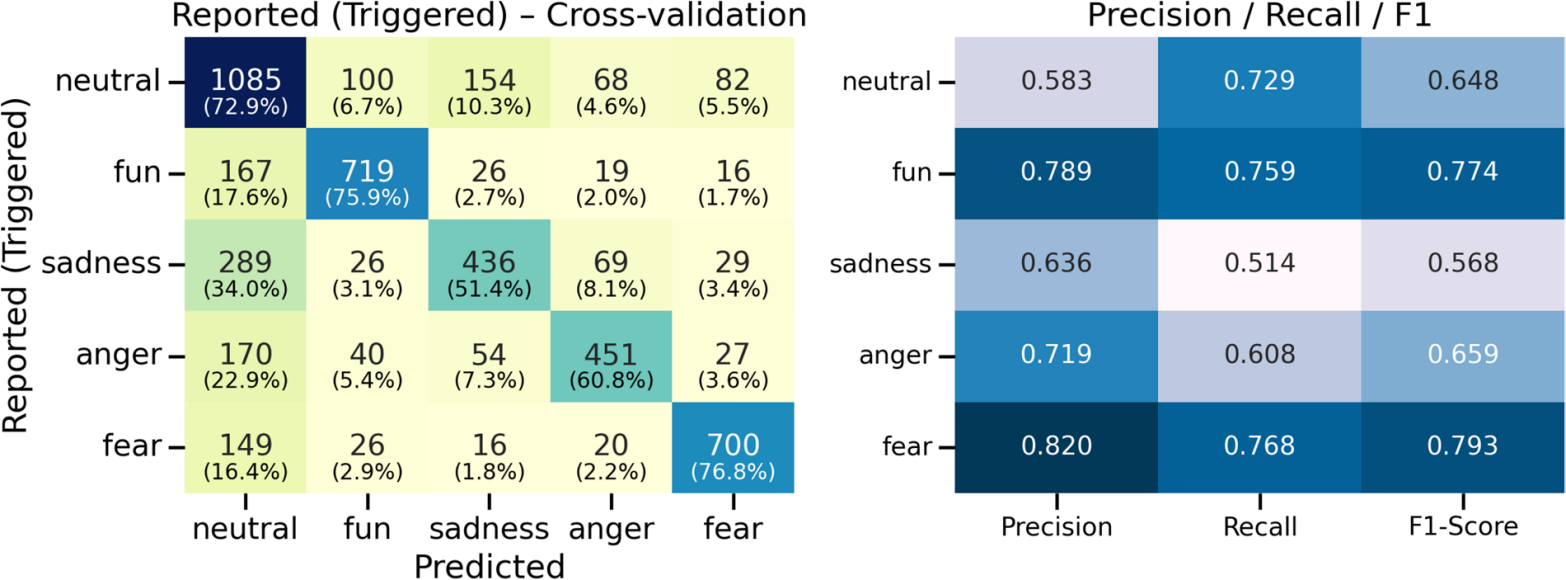
Cross-validation results using LightGBM for biosignal-based emotion classification.

Using these features, a light gradient boosting machine (LightGBM) classifier was trained to classify five discrete emotional states (neutral, fun, sadness, anger, fear). The model achieved an accuracy of 68.2% and a Cohen’s *κ* of 0.590. As shown in Fig. 8, high-arousal emotions (fun, anger, fear) exhibited precision values above 0.710, whereas lower-arousal states (neutral, sadness) showed precision values of 0.581 – 0.622. This indicates differential discriminability across emotional categories. These findings suggest that multimodal biosignals, particularly EEG and peripheral features, contain informative patterns for decoding affective states, thereby providing supporting evidence that the stimuli successfully elicit distinguishable physiological responses.

## Usage Notes

### Figshare repository

The dataset preprocessing and analysis code associated with this paper has been uploaded to 10.6084/m9.figshare.28748696.v6^32^. The codebase consists of one Jupyter Notebook and several standalone Python scripts. The Jupyter Notebook file ‘2025_Kitech_Emotion_Data_Code.ipynb’ contains code for preprocessing and analyzing the dataset introduced in this study. The script iteratively processes data for 108 subjects, calculating statistical metrics to validate emotion classification consistency. Specifically, the code computes Cohen’s *κ* and overall accuracy by comparing intended versus reported emotions, and visualizes the results as a confusion matrix heatmap. It relies on the following packages and versions: numpy (2.3.2), pandas (2.3.1), scikit-learn (1.7.1), xgboost (3.0.4), lightgbm (4.6.0), matplotlib (3.10.5), and seaborn (0.13.2). Additional scripts include ‘compute_snr.py’ for calculating the SNR, ‘compute_snr_statistics.py’ for computing SNR statistics, and ‘compute_raw_statistics.py’ for generating statistics from the raw data.

### Limitations

Firstly, air conditioning was used during the experiments to maintain a stable temperature; however, it was not fully effective at controlling humidity. Variations in humidity can influence physiological signals such as skin conductance, potentially introducing noise or variability in the measurements. Despite this limitation, the presence of air conditioning reduced extreme fluctuations in environmental conditions compared to an uncontrolled setting, ensuring that the resulting dataset remains valuable and suitable for affective computing research. For future data collection, air conditioning may be complemented with a dehumidifier to achieve more consistent control of both temperature and humidity.

Secondly, participants were instructed not to consume caffeine on the day of the experiment and to abstain from alcohol the day before. However, there was no objective way to verify compliance. In addition, no restrictions were imposed on food intake prior to the experiment, even though pre-experimental dietary control would have been desirable. One candidate was, in fact, excluded from participation after self-reporting alcohol consumption during the briefing session. Although such restrictions may not have been strictly enforced, participants were explicitly instructed regarding caffeine and alcohol avoidance, and it is likely that most participants complied. Therefore, the dataset retains its value. For future data collection, stricter protocols will be adopted, following established guidelines—for example, no meals within 2 hours before the experiment, no caffeine within 6 hours, and no alcohol within 24 hours. Furthermore, objective verification such as breath alcohol testing will be implemented to ensure adherence.

## Data Availability

The dataset is available at 10.6084/m9.figshare.28748696.v6.

## Code availability

The code for calculating raw biosignal statistics, computing the SNRs of biosignals, calculating statistics of the SNRs, performing preprocessing, and conducting LightGBM-based emotion classification is available at: 10.6084/m9.figshare.28748696.v6.
